# Altered functional connectivity in binge eating disorder and bulimia nervosa: A resting‐state fMRI study

**DOI:** 10.1002/brb3.1207

**Published:** 2019-01-15

**Authors:** Marion A. Stopyra, Joe J. Simon, Mandy Skunde, Stephan Walther, Martin Bendszus, Wolfgang Herzog, Hans‐Christoph Friederich

**Affiliations:** ^1^ Department of General Internal Medicine and Psychosomatics, Centre for Psychosocial Medicine University Hospital Heidelberg Heidelberg Germany; ^2^ Psychological Institute Heidelberg University Heidelberg Germany; ^3^ Department of Psychosomatic Medicine and Psychotherapy, Medical Faculty Heinrich‐Heine‐University Düsseldorf Düsseldorf Germany; ^4^ Department of General Adult Psychiatry, Centre for Psychosocial Medicine University Hospital Heidelberg Heidelberg Heidelberg Germany; ^5^ Department of Neuroradiology University Hospital Heidelberg Heidelberg Germany

**Keywords:** binge eating disorder, bulimia nervosa, functional connectivity, resting‐state fMRI

## Abstract

**Introduction:**

The etiology of bulimic‐type eating (BTE) disorders such as binge eating disorder (BED) and bulimia nervosa (BN) is still largely unknown. Brain networks subserving the processing of rewards, emotions, and cognitive control seem to play a crucial role in the development and maintenance of eating disorders. Therefore, further investigations into the neurobiological underpinnings are needed to discern abnormal connectivity patterns in BTE disorders.

**Methods:**

The present study aimed to investigate functional as well as seed‐based connectivity within well‐defined brain networks. Twenty‐seven individuals with BED, 29 individuals with BN, 28 overweight, and 30 normal‐weight control participants matched by age, gender, and education underwent resting‐state functional magnetic resonance imaging. Functional connectivity was assessed by spatial group independent component analysis and a seed‐based correlation approach by examining the default mode network (DMN), salience network (SN), and executive network (EN).

**Results:**

Group comparisons revealed that BTE disorder patients exhibit aberrant functional connectivity in the dorsal anterior cingulate cortex (dACC) within the SN, as well as in the medial prefrontal cortex within the DMN. Furthermore, BED and BN groups differed from each other in functional connectivity within each network. Seed‐based correlational analysis revealed stronger synchronous dACC‐retrosplenial cortex activity in the BN group.

**Conclusion:**

Our findings demonstrate abnormalities in brain networks involved in salience attribution, self‐referential processing, and cognitive control in bulimic‐type eating disorders. Together with our observation of functional connectivity differences between BED and BN, this study offers a differentiated account of both similarities and differences regarding brain connectivity in BED and BN.

## 1 INTRODUCTION

Binge eating disorder (BED) and bulimia nervosa (BN) are eating disorders characterized by recurrent episodes of binge eating and a loss of control during binging. Individuals with BN counteract binge eating to prevent weight gain by compensatory behaviors such as self‐induced vomiting or laxative/diuretic abuse, whereas individuals with BED do not exhibit recurrent compensatory behaviors causing overweight and obesity (American Psychiatric Association, 2013; Sim et al., 2010).

The neurobiological mechanisms involved in the development and maintenance of binge‐type eating disorders are still unclear. To date, most imaging studies examined task‐dependent brain activity. However, little is known about impairments of functional brain networks in individuals with BED and BN. Contemporary research has begun to emphasize the role of interactions and connections between spatially distributed brain areas. For example, functional connectivity is inferred from temporal correlations of neural activation patterns which are thought to represent common functional properties (Deco, Jirsa, & McIntosh, 2013; van den Heuvel & Hulshoff Pol, 2010; Watts & Strogatz, 1998). A number of functional connectivity alterations have been found in patients with BN. The cerebellum, a region crucial for feeding behavior, appetite, and emotional regulation (Mahler, Guastavino, Jacquart, & Strazielle, 1993; Zhu & Wang, 2008) has been found to exhibit hyperconnectivity with the dorsal anterior cingulate cortex (dACC) and the anterior insula in BN (Amianto et al., 2013). The hyperconnectivity with the anterior cingulate cortex (ACC) is in line with the findings of impulse control difficulties present in BN (Joos et al., 2011). Possibly, the dysfunctional ACC activity contributes to a lack of self‐regulatory control which in turn results in impulsive behavior. The hyperconnectivity with the insula might be indicative of distortions in body awareness or a sign of altered taste processing, which both underlie the psychopathology of BN (Mohr et al., 2011; Monteleone et al., 2017). Moreover, increased coupling between the dACC as a seed region and the orbitofrontal cortex as well as precuneus has been demonstrated in BN (Lee et al., 2014). These brain regions have been implicated in self‐referential processing which involves monitoring one's external environment, physical appearance, and emotional state (Davey, Pujol, & Harrison, 2016), indicating that individuals with BN exhibit excessive self‐introspection contributing to the preoccupation of body image which is in line with behavioral data (Benninghoven, Raykowski, Solzbacher, Kunzendorf, & Jantschek, 2007). However, relatively little is known about functional connectivity networks in BED (Kessler, Hutson, Herman, & Potenza, 2016). A recent study by Baek, Morris, Kundu, and Voon (2017) did not find global network differences between obese individuals with BED and obese individuals without BED, possibly due to the small sample size. Therefore, they merged both groups and compared the obese group with normal‐weight healthy control participants. Similar to previous reports who observed impairments in the salience network in participants with obesity (García‐García et al., 2013), the obese group exhibited decreased functional connectivity in cortico‐striatal and cortico‐thalamic networks, further corroborating the importance of dopaminergic processing in the development and maintenance of obesity (Volkow, Wang, & Baler, 2011).

Taken together, preexisting findings indicate a number of neural network alterations in individuals with BN, BED, and overweight. Impairments in functional connectivity appear to be a crucial aspect in the neuropathology of eating disorders and overeating. However, a number of open questions still remain. Specifically, since both BED and BN are characterized by compulsive overeating, investigating their shared neural alterations during resting‐state fMRI as well as how the neurobiological mechanisms underlying these eating disorders differ from weight‐matched healthy control participants is of specific importance. Therefore, the goals of this study were to identify differences in resting‐state networks between individuals with BN, BED, and their respective control groups.

Resting‐state functional magnetic resonance imaging (rs‐fMRI) has become fundamental for the investigation of task‐unrelated spontaneous blood oxygen level‐dependent (BOLD) signal fluctuations when a participant is not performing an explicit task (Lee, Smyser, & Shimony, 2013). The present study examined three resting‐state networks involving brain areas which have been previously implicated in food and reward processing. The default mode network (DMN) involves a functionally connected network of brain regions such as the posterior cingulate cortex (PCC), medial prefrontal cortex, and temporal cortices and is thought to be implicated in self‐referential processing which involves monitoring the external environment as well as physical and emotional states (Davey et al., 2016). Previous studies found increased activity in the DMN in obese compared to lean individuals (Tregellas et al., 2011). The second network, the salience network (SN) including the insula, dorsal ACC, and other subcortical structures, is involved in emotional arousal, food, and reward processing (Seeley et al., 2007). A third network, the executive network (EN) includes the dorsolateral prefrontal cortex (DLPFC) and parietal cortices and is involved in inhibitory processes like termination of food‐consumption (DelParigi, Chen, Salbe, Reiman, & Tataranni, 2005). Finally, we performed a seed‐based functional connectivity analysis to identify aberrations in connectivity between disorder‐related brain regions. The region of interest (dACC) was chosen based on the observed difference between the BN and BED group.

## 2 MATERIALS AND METHODS

### 2.1 Participants

In total, 120 participants (30 patients with BED, 30 patients with BN, 30 normal‐weight controls [ConBN], and 30 overweight controls [ConBED]) were enrolled in the present study. The overweight and normal‐weight control group were matched in age, education, and BMI to the BED and BN group (Table 1). All participants were female, except for four male participants each in the BED as well as the ConBED group. All participants were right‐handed and were screened for medical and psychiatric disorders using the Structured Clinical Interview for DSM‐IV (SCID, Wittchen, Zaudig, & Fydrich, 1997). The SCID was also used to classify patients according to DSM‐IV criteria into the BED and BN group. Exclusion criteria for all participants were neurological or psychiatric disorders, brain injury, substance dependence or abuse, smoking, pregnancy, claustrophobia, or any implants contraindicated in MRI. Patients with BED or BN did not take medication besides antidepressants (four participants in the BED and seven participants in the BN group were receiving antidepressant medications). Participants filled out a number of self‐report and demographic questionnaires and were asked about their eating and dieting behaviors. Specifically, all participants filled out the Beck Depression Inventory (BDI, Hautzinger, Keller, & Kühner, 2006), the Dutch Eating Behavior Questionnaire (DEBQ, Grunert, 1989), and the State and Trait version of the General Food Craving Questionnaire (G‐FCQ, Nijs, Franken, & Muris, 2007). Six participants were excluded (none of them were males) due to motion artifacts and technical issues, resulting in a final sample of 29 individuals with BN, 27 individuals with BED, 30 normal‐weight controls, and 28 overweight controls. Individuals with BED and BN were recruited from our ward and outpatient clinic, while the ConBN group and ConBED group were recruited by flyer advertisements. The local Ethics committee approved the study, and it is in accordance with the ethical standards of the Declaration of Helsinki in 1975 as revised in 2008. All participants gave informed written consent prior to participation.

**Table 1:** Demographics and clinical characteristics of control and patient group

	BED patients (*n* = 27)		BED controls (*n* = 28)		*p*	BN patients (*n* = 29)		BN controls (*n* = 30)		*p*
	Mean	*SD*	Mean	*SD*		Mean	*SD*	Mean	*SD*	
Age (years)**	38.39	13.06	39.40	10.48	0.76	27.45	10.55	26.86	6.59	0.80
Body mass index**	32.64	4.13	33.58	4.54	0.43	21.33	2.99	21.85	1.80	0.43
Education (years)	12.04	1.76	12.00	2.02	0.94	12.66	1.40	12.733	0.83	0.80
Verbal intelligence (MWT‐B Score)	109.41	15.16	106.29	10.20	0.377	105.38	12.27	106.4	13.82	0.766
Beck Depression Inventory	22.07	11.09	7.93	7.23	<0.001	25.33	12.31	3.67	3.11	<0.001
DEBQ – total	74.93	15.47	49.21	14.42	<0.001	82.52	16.39	37.13	12.74	<0.001
DEBQ – restrained eating**	19.89	6.50	15.89	7.41	0.042	28.83	7.63	11.53	8.11	<0.001
DEBQ – emotional eating	28.04	9.18	13.57	9.30	<0.001	28.52	7.94	7.13	4.73	<0.001
DEBQ – external eating	27.00	7.27	19.75	6.75	<0.001	25.17	7.18	28.47	6.58	<0.001
Food craving questionnaire ‐ state	41.96	14.86	32.54	12.72	0.015	39.55	13.49	32.73	10.19	0.033
Food craving questionnaire ‐ trait	83.46	18.85	56.07	17.79	<0.001	83.59	15.71	44.73	11.72	<0.001
Binge eating per week*	2.56	1.52	‐	‐		3.83	2.58	‐	‐	

* Significant differences between BED and BN group at *p *< 0.05.

** Significant differences between BED and BN group at *p *< 0.001.

### 2.2 Resting‐state data analysis

#### 2.2.1 Data acquisition

Images were collected using a Tim Trio 3‐T whole‐body MR scanner (Siemens Medical Solutions, Erlangen, Germany) equipped with a standard 32‐channel head coil. Prior to performing an event‐related reward task (results are reported elsewhere (Simon et al., 2016; Skunde et al., 2016)), every participant underwent a resting‐state MR acquisition lasting 5.4 min. A total of 162 images composed of 30 oblique interleaved slices with an 1‐mm interslice gap were collected using a T2*‐sensitive single‐shot EPI sequence. Slices were acquired parallel to the AC‐PC axis with the following parameters: TR = 2,000 ms, TE = 30 ms (which resulted in an in‐plane resolution of 3 × 3 × 4 mm^3^), flip angle = 80°, and field of view = 192 × 192 mm. Furthermore, high‐resolution T1 MPRAGE anatomical images were acquired (192 slices, voxel size 1 × 1 × 1 mm^3^, TR = 1,570 ms, TE = 2.63 ms, flip angle = 9°) for anatomical reference. Participants were instructed to keep still with their eyes closed, not to think of anything in particular, and not to fall asleep.

#### 2.2.2 Data preprocessing

Resting‐state fMRI data were preprocessed and analyzed with Statistical Parametric Mapping (RRID:SCR_007037; SPM8, Wellcome Department of Imaging Neuroscience, London, UK; http://www.fil.ion.ucl.ac.uk/spm/software/spm8/). To account for magnetic field equilibration, four volumes from the start of each functional run were excluded from the analysis. Prior to the statistical analysis, functional images were preprocessed by performing slice‐time correction, realignment, and unwarping to correct for susceptibility‐by‐movement interactions, coregistration with mean T2* images, and segmentation, normalization to MNI‐space using the transformation parameters from the segmentation, resulting in a voxel size of 3 × 3 × 4 mm^3^, and finally images were smoothed using an 8‐mm full‐width half‐maximum isotropic Gaussian kernel. To evaluate the quality of our rs‐fMRI data, we performed a manual inspection of all images (no participant exceeded ± 2.5 mm translational movement or ±2 degrees of rotation over the entire 160 data points) and used the TsDiffAna toolbox (RRID:SCR_016656; http://www.fil.ion.ucl.ac.uk/spm/ext/#TSDiffAna) to control for data artifacts. Furthermore, we employed “Artifact Detection Tools” (ART; RRID:SCR_005994; http://www.nitrc.org/projects/artifact_detect) to identify problematic time points during the scan. An image was identified as an outlier if the head displacement exceeded a threshold of 2 mm normalized movement in any direction or if the global mean intensity threshold in the image exceeded three standard deviations from the mean image intensity for the entire scan. The mean amount of motion outliers was 0.32 ± 1.17, and the intensity outliers had a mean of 4.91 ± 7.74. Importantly, the four groups did not differ regarding the number of intensity (*t*(53) = 1.04, *p* = 0.305 for overweight controls and BED group and *t*(57) = −1.48, *p* = 0.145 for the BN and normal‐weight group) and motion outliers (*t*(53) = 1.99, *p* = 0.051 and *t*(57) = 0.83, *p* = 0.411 for the BED and BN group, respectively).

#### 2.2.3 Independent component analysis

We performed a spatial group independent component analysis (ICA, Calhoun, Adali, Pearlson, & Pekar, 2001) using the “Group ICA fMRI Toolbox” (GIFT; RRID:SCR_001953; http://mialab.mrn.org/software/) to identify temporally coherent resting‐state networks. The dimensionality of the preprocessed rs‐fMRI data from each subject was reduced through principal component analysis. Using the high‐model order ICA approach proposed by Allen et al. (2011), the data were then decomposed into 75 spatial independent components based on the infomax‐algorithm (Bell & Sejnowski, 1995) implemented in GIFT. We performed 20 ICA (ICASSO) to ensure stability of the estimated components. For each subject, component spatial maps were reconstructed (using GICA3, Erhardt et al., 2011) and converted to *z*‐values.

#### 2.2.4 Component selection

**Figure 1:**
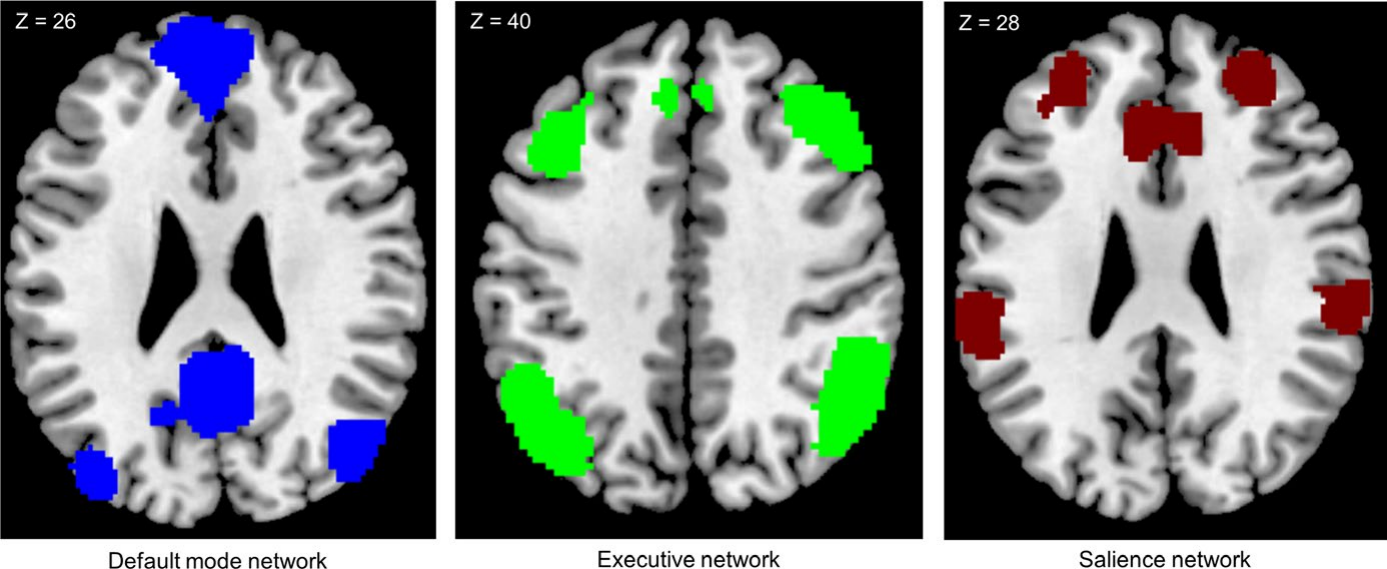
Employed masks for the three different intrinsic connectivity networks: the default mode network, the executive network, and the salience network

We employed a spatial correlation to identify three resting‐state networks from the resulting components which have been previously implicated in food and reward processing. We used anatomical masks based on the “Willard” atlas (RRID:SCR_014756; Richiardi et al., 2015) taken from https://findlab.stanford.edu/functional_ROIs.html. The DMN mask was composed of the medial prefrontal cortex, posterior cingulate cortex, retrosplenial cortex, and the medial temporal lobe. The SN mask was composed of the anterior and posterior insula, dorsal anterior cingulate cortex, Thalamus, Caudatus, and Putamen. The EN mask included the left and right dorsolateral prefrontal cortex as well as bilateral parietal cortex (see Figure 1). We chose the component with the highest correlation with the respective template mask.

#### 2.2.5 Differences in functional connectivity between groups

To assess differences between groups in functional connectivity within the different networks of interest, individual component images were included in a random effect analysis and compared using a two‐sample *t* test with age and depressive symptoms (assessed using the BDI) as covariates of no interest. We employed a cluster defining threshold of *p* < 0.001 uncorrected (cluster size *k* > 30) and report results significant at a family‐wise error‐corrected cluster level threshold of *p* < 0.05. Results were masked using the respective anatomical masks (DMN: *r* = 0.344, SN: *r* = 0.345, EN: *r* = 0.138). Group statistics were visualized using MRIcron (Chris Rorden, version 4, April 2011, RRID:SCR_002403).

#### 2.2.6 Seed‐to‐voxel correlational analyses

To assess functional connectivity of the dACC, we performed a seed‐based functional connectivity analysis using the functional connectivity (CONN) toolbox (RRID:SCR_009550) version 16.b (Whitfield‐Gabrieli & Nieto‐Castanon, 2012, http://www.nitrc.org/projects/conn) and SPM8. The dACC was chosen as BED, and BN patients showed differences within this region in ICA‐analyses compared to their respective control groups (see Results for details). Furthermore, the dACC is considered as a core region of the salience network (Seeley et al., 2007). We defined an anatomical region of interest (ROI) in the bilateral dACC using the WFU Pickatlas (RRID:SCR_007378; http://fmri.wfubmc.edu/software/pickatlas) and the MarsBar toolbox (RRID:SCR_009605; http://marsbar.sourceforge.net/). Based on a previous publication (Cascio, Konrath, & Falk, 2015), the ROI was created by combining Brodmann areas 24 and 32, as well as the anterior, middle, and posterior cingulate masks from the AAL atlas. All masks were dilated to 2 mm, and Brodmann areas 8 and 9 were subtracted from the ROI. Finally, the ROI was restricted to a bounding box of *x* = −16 to 16, *y* = 0 to 33, *z* = 6 to 52.

In a first step, functional images were preprocessed using the steps described above with the addition of artifact scrubbing (using the ART toolbox as implemented in CONN) and band‐pass filtering of 0.01–0.1 Hz as recommended by the CONN toolbox. In a second step, the component‐based noise correction method (CompCor, Behzadi, Restom, Liau, & Liu, 2007) was employed to correct for physiological and other noise sources. Specifically, signal noise from white matter and cerebrospinal fluid was extracted by principal component analysis and included in the model as covariates of no interest in addition to motion parameters and outliers. First‐level analysis was performed using a general linear model to assess significant BOLD signal correlation with respect to time between the dACC seed and each voxel. The resulting correlation coefficients were converted to *z*‐scores using Fisher's *Z* transformation. Second‐level analysis was performed using a two‐sample *t* test to compare groups, with a cluster defining threshold of *p* < 0.001 uncorrected (cluster size *k* > 30). Results significant at a family‐wise error‐corrected cluster level threshold of *p* < 0.05 are reported.

#### 2.2.7 Correlational analysis

To assess the relation between degree of illness and connectivity of the dACC, we extracted eigenvariates from the retrosplenial cortex during the *BN > BED* contrast and from the precentral gyrus during the *BED* > *BN* contrast (the observed clusters of connectivity profiles during both contrasts were used as masks). Spearman correlations were used to test for the association between binge frequency and functional connectivity between the seed and target region.

## 3 RESULTS

### 3.1 Participants

As shown in Table 1, there were no significant group differences regarding age, BMI, education years, and verbal intelligence between the patient groups and their respective control group. However, both BED and BN patients displayed higher levels of depressive symptoms than controls as well as higher scores on the DEBQ‐scale and on the food craving questionnaire (all *P*s < 0.033, see Table 1). Finally, when comparing the BED and BN groups, there were significant differences in age, BMI, and the frequency of binges per week (all *P*s < 0.029). No significant group differences were found in verbal intelligence, education, food craving scores, and the DEBQ, with the exception of the subscale “restraint eating,” where BN patients scored significantly higher compared to the BED group (*p* < 0.001, see Table 1).

### 3.2 Independent component analysis

**Figure 2:**
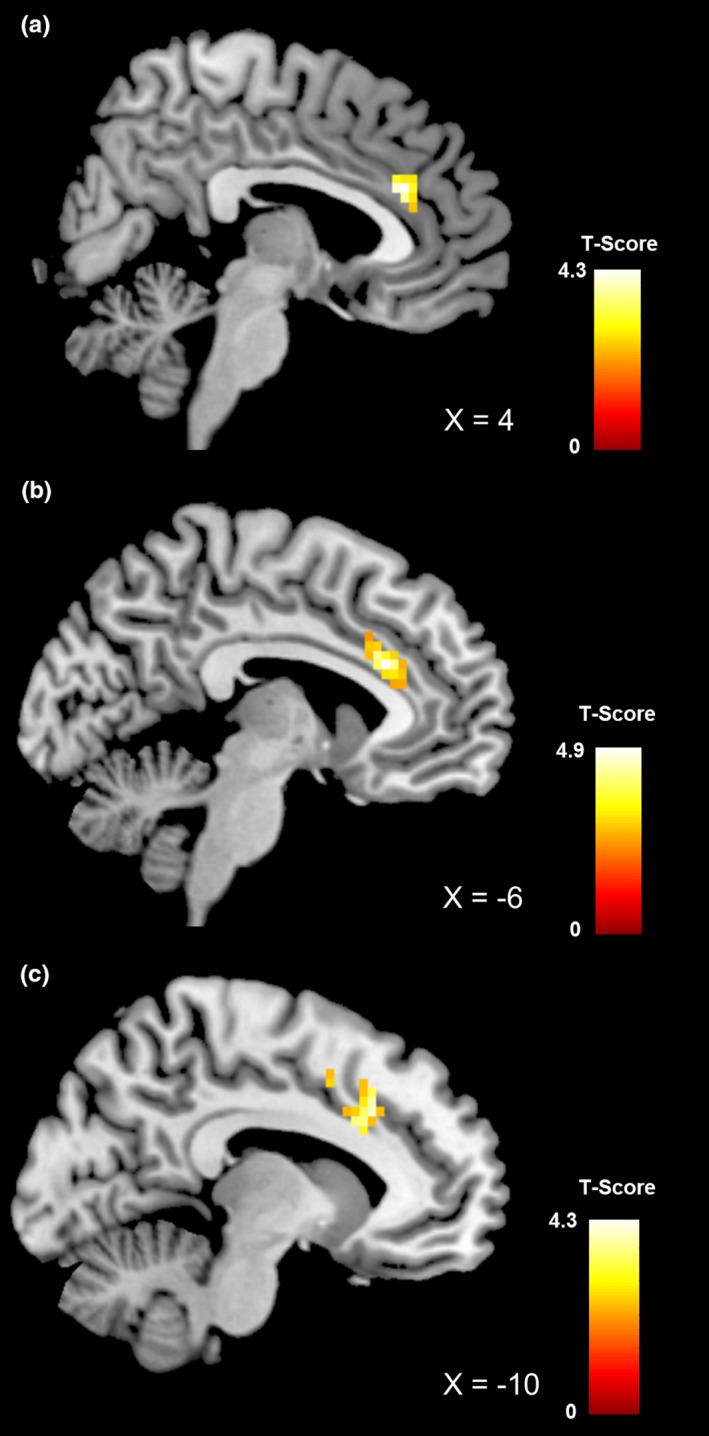
Brain region showing significant differences in functional connectivity of the salience network between healthy obese control participants and patients with binge eating disorder (a), between healthy normal‐weight control participants and patients with bulimia nervosa (b), and between patients with binge eating disorder and patients with bulimia nervosa (c). All results are cluster‐wise *p* < 0.05 corrected; color bar represents *t*‐values

**Figure 3:**
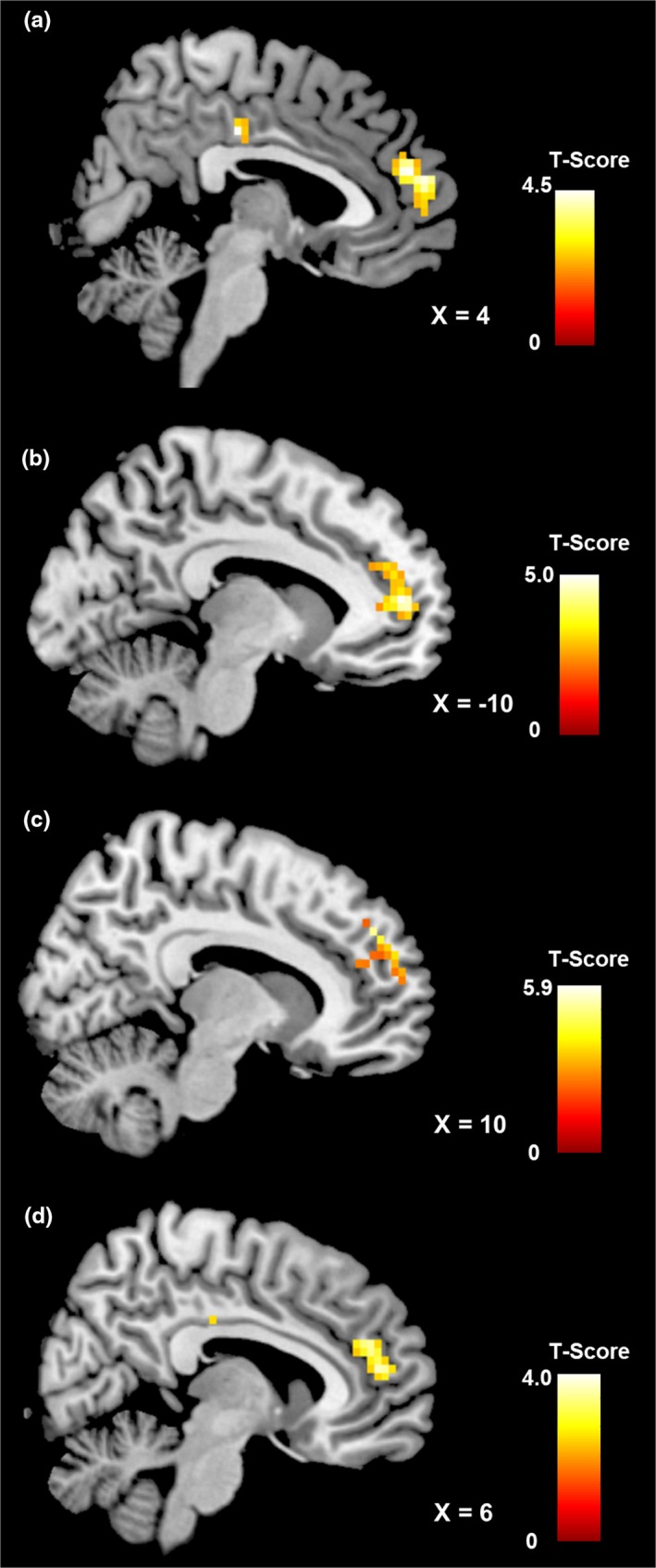
Brain region showing significant differences in functional connectivity of the default mode network between healthy obese control participants and patients with binge eating disorder (a), between healthy normal‐weight control participants and patients with bulimia nervosa (b), between patients with bulimia nervosa and healthy normal‐weight participants (c), and between patients with bulimia nervosa and patients with binge eating disorder (d). All results are cluster‐wise *p* < 0.05 corrected; color bar represents *t*‐values

To assess differences between groups, we compared functional connectivity within the three identified networks of interest (see Table 2, Figure 1). For the *salience network*, we observed a significant increase in functional connectivity in the anterior portion of the dorsal cingulate cortex in both the normal‐ and overweight control groups when compared to the BN and BED groups, respectively. Interestingly, when comparing patient groups, we also observed a significant difference in the cingulate cortex; patients with BED displayed stronger connectivity in the posterior part of the dorsal cingulate cortex than BN patients (Figure 2). In order to compare the healthy control groups with bulimic‐type eating disorders, we pooled the four groups into a BTE group (i.e., BED and BN) and a control group (i.e., ConBN and ConBED). Similar to the separate group results, we observed increased connectivity in the dorsal anterior cingulate cortex in the control group when compared to the BTE group (see Supporting information Table S1). For the *default mode network*, we observed a stronger connectivity within the dorsal medial prefrontal cortex for the BN group when compared to both their respective control group and the BED group. Furthermore, the BED group displayed lower connectivity in this region when compared to their respective control group. The BN group showed increased activity in a lateral portion of the ventral medial prefrontal cortex when compared to their respective control group (Figure 3). Finally, although we observed no differences between the patient groups and their respective control groups for the *executive network*, we found increased connectivity in the middle frontal gyrus and angular gyrus in the BN group when compared to the BED group. The pooled control group showed increased functional connectivity in the inferior parietal cortex when compared to the BTE group (see Supporting information Table S1). It has to be noted that the comparison between groups was performed with the inclusion of depressive symptoms as covariates of no interest. Since there was a significant difference in number of binges between patient groups, we repeated the analysis with the inclusion of number of binges as covariate of no interest and found that this did not affect the results. Furthermore, there was a significant difference in both BMI and age between patient groups. To account for this, we compared the contrast BN versus BED with the contrast ConBN versus ConBED, contrasted the BN group with the ConBED group and the BED group with the ConBN group. Since control groups were matched for age and BMI, they also differed regarding those variables, allowing us to assess the specificity of our results. In a first step, we visually compared the results from all three networks. In a second step, we performed a disjunction analysis to obtain an objective measure for similarities. Since the contrasts were derived from different statistical models, the thresholded statistical maps were binarized and then subtracted from each other using the ImCalc function (as implemented in SPM8). Both, the visual analysis and the disjunction analysis, confirmed that our observations were not simply due to age and BMI, since we did not observe overlaps between the results.

**Table 2:** Results obtained from the independent component analysis

Group/brain regions	Hemisphere	BA	*x*	*y*	*z*	No. of voxels	*p‐*value	*t‐*value
Salience network								
ConBED > BED								
Medial dorsal CC (anterior)	R	32	3	33	24	33	0.030	4.37
ConBN > BN								
Medial dorsal CC (anterior)	L	24	−6	27	24	43	0.001	4.95
BED > BN								
Medial dorsal CC (posterior)	L	32	−9	24	34	38	0.020	4.36
DMN								
ConBED > BED								
Dorsal medial prefrontal cortex	R	9	3	51	20	51	0.003	4.55
ConBN > BN								
Ventral medial prefrontal cortex	L		−12	54	7	56	0.003	5.01
BN > ConBN								
Dorsal medial prefrontal cortex	R	9	6	51	31	50	0.005	5.99
BN > BED								
Dorsal medial prefrontal cortex	R	9	3	45	24	54	0.003	4.03
Executive network								
BN > BED								
Middle frontal gyrus	L	9	−39	18	37	36	0.002	5.31
Angular gyrus	L	39	−45	−66	34	40	0.001	4.32

Note

Results were FWE cluster‐corrected (voxel *p*  < 0.001; cluster > 30 voxels, *p *< 0.05).

Unlisted contrasts indicate a lack of significant results.

BA: Brodmann area; L: left; R: right; CC: cingulate cortex.

Correction added on 23 January 2019, after first online publication: Tables 2 and 3 have been interchanged.

### 3.3 Seed‐based functional connectivity analysis

**Figure 4:**
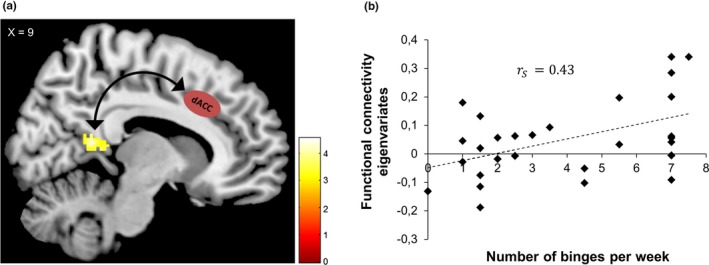
Functional connectivity between dACC as a seed region and retrosplenial cortex in patients with bulimia nervosa (a) and correlation between eigenvariates extracted from the retrosplenial cortex and the frequency of binges experienced by patients with bulimia nervosa (b)

No significant functional connectivity differences between the dACC and the rest of the brain were found when comparing the BN group with their respective control group. The BED group displayed significantly increased connectivity in the right cerebellum and the right lingual gyrus when compared to the ConBED group. When comparing the BN group with the BED group, a correlational seed‐to‐voxel analysis revealed that the BN group exhibited significantly stronger functional connectivity between the dACC and bilateral retrosplenial cortex (BA 29), respectively (Figure 4a). The BED group compared to the BN group demonstrated increased synchronized activities between the dACC and the pre‐ and postcentral gyrus/somatosensory cortex (see Table 3). To control for the influence of BMI and age, we employed the same method as detailed in the results of the independent component analysis, which did not reveal any overlap with the results of the seed‐to‐voxel analysis.

**Table 3:** Functional connectivity differences in the dACC between groups

Group/brain regions	Hemisphere	BA	*x*	*y*	*z*	No. of voxels	*p‐*value	*t‐*value
BED > ConBED								
Cerebellum	R		4	−54	−44	148	0.002	4.87
Lingual gyrus	R	18	0	−86	−14	187	0.001	4.09
BN > BED								
Retrosplenial cortex	L	29/30	−8	−44	20	258	<0.001	4.36
	R		8	−48	14			
BED > BN								
Precentral gyrus	L	6	−48	−4	−34	190	0.001	4.64
Postcentral gyrus	R	6	50	−10	32	177	0.001	4.38
SMA	L		−8	−20	50	292	<0.001	4.26
ConBED > ConBN								
Middle frontal gyrus	L	6	−20	−4	44	423	<0.001	5.60
Postcentral gyrus	R	4	34	−30	52	2090	<0.001	4.80
Precentral gyrus	R	6	24	0	50	198	0.001	4.26
ConBN > ConBED								
Cerebellum	R		4	−54	−44	721	<0.001	6.73
Thalamus	R		12	−30	2	410	<0.001	5.33
Calcarine sulcus	R	18	2	24	18	407	<0.001	4.96
Middle occipital gyrus	L	19	34	−94	10	1,032	<0.001	4.34

Note

Results significant at voxel level *p *< 0.001; cluster > 30 voxels, *p *< 0.05.

Unlisted contrasts indicate a lack of significant results.

BA: Brodmann area; L: left; R: right; SMA: supplementary motor area.

Finally, post hoc analyses of the parameter estimates which were extracted from the retrosplenial cortex in the BN group revealed a positive correlation with the amount of binge eating episodes per week (Spearman's ρ = 0.43, *p* = 0.022, Figure 4b). In contrast, there was no significant correlation between the number of binges and the synchronous dACC‐somatosensory cortex activation for the BED group. Furthermore, there was no significant interaction between BMI and parameter estimates from the retrosplenial cortex in the BN group (Spearman's ρ = −0.11, *p* = 0.576). Importantly, when including age as a covariate of no interest in our seed‐based connectivity analysis, the observed activation in the retrosplenial cortex failed to reach our pre‐defined significance level.

## 4 DISCUSSION

The present study used resting‐state fMRI to examine functional connectivity changes in the DMN, the SN, and the EN in individuals with bulimia nervosa and binge eating disorder, as well as obese and normal‐weight participants. We observed differences in connectivity between groups, notably in the salience and default mode network. Furthermore, seed‐based functional connectivity results showed a stronger dACC‐retrosplenial coupling in individuals with BN and an increased dACC‐somatosensory cortex coupling in BED. The dACC‐retrosplenial cortex interaction correlated with the quantity of weekly binges. Our results offer a differentiated account of the role of these brain networks in BTE disorders.

When analyzing group differences in the SN, we observed connectivity differences in the dACC for both patients with BED and BN. Specifically, healthy controls displayed a cluster of increased connectivity in the anterior portion of the dACC. These findings are in line with previous studies indicating structural and functional connectivity abnormalities of the SN in individuals with eating disorders (Friederich et al., 2012; Peters, Dunlop, & Downar, 2016; Schäfer, Vaitl, & Schienle, 2010). This network is essential for the processing of personally salient stimuli and involved in emotional arousal, food, and reward processing (DelParigi et al., 2005) and might therefore play an essential role in the psychopathology of BTE disorders. Furthermore, the ACC has been consistently found to be associated with both BED and BN (Geliebter, Benson, Pantazatos, Hirsch, & Carnell, 2016; Schienle, Schäfer, Hermann, & Vaitl, 2009; Seitz et al., 2016). Especially the dorsal part of the ACC has been described as an important hub of the SN (Seeley et al., 2007), our results therefore suggest alterations in salience processing circuits as a possible candidate for an underlying trait marker of BTE disorders. It should be noted that these findings are spread across multiple Brodmann areas (i.e., 24 and 32) of the anterior portion of the dACC. Little research has been conducted on the functional segregation of these two areas; however, area 24 and 32 seem to be functionally heterogeneous. Both areas are significantly associated with the conscious experience of emotions and cognitive processes. Area 32 was particularly linked to fear and memory processes, while area 24 was specifically related to sadness, the sense of tasting, and interoceptive processes (Palomero‐Gallagher et al., 2015).

When comparing patient groups, patients with BED displayed increased connectivity in the posterior part of the dACC. Although the diminished SN connectivity is observable in both groups, we found it to be more pronounced for BN participants. To further investigate upon this, an additional connectivity analysis with the dACC as a seed region was performed which demonstrated greater synchronous activity between the dACC as a seed region and the bilateral retrosplenial cortex in BN, whereas the BED group showed greater stronger functional connectivity between the dACC and the somatosensory cortex. The retrosplenial cortex has increasingly received attention in neuroimaging studies in contexts of self‐referential processing such as perspective‐taking and self‐descriptions and plays a central role in the DMN (Cavanna & Trimble, 2006). Our findings are in accordance with Lee et al (2014) who demonstrated synchronous dACC‐retrosplenial cortex connectivity in patients with BN and AN. Furthermore, in a previous analysis with the same sample using a food incentive delay task, we observed increased activation in regions associated with self‐referential processing during the receipt of food‐related rewards (Simon et al., 2016). Taken together with our observation of increased restrained eating in the BN group, it is plausible that the stronger connectivity between the retrosplenial cortex and dACC is associated with disorder‐specific rumination on eating, weight, and body shape in individuals with bulimia nervosa. This result is further supported by the positive correlation between the dACC‐retrosplenial cortex interaction and the number of binges experienced by BN patients, as the frequency of binge‐purging behavior might be an indicator of the degree of the illness. Our finding is supported by an extensive review which highlights the positive relationship between neurobiological abnormalities and frequency of binge eating (Donnelly et al., 2018).

Furthermore, we also observed group differences in the DMN; patients with BN displayed increased connectivity in the anterior part of the DMN (medial prefrontal cortex; mPFC) when compared with matched controls and patients with BED. The mPFC has been repeatedly associated with self‐referential thoughts (Johnson et al., 2002; Macrae, Moran, Heatherton, Banfield, & Kelley, 2004). A recent study by Seitz et al (2016) demonstrated a similar finding with BN patients exhibiting a failure to deactivate the DMN. This has been interpreted as a constant preoccupation with food or body image‐related thoughts. This interpretation is in line with behavioral findings indicating a preoccupation of bulimic women with their physical and social self (Striegel‐Moore, Silberstein, & Rodin, 1993). The lack of purging observed in BED might therefore be related to the decreased preoccupation with food or body image‐related thoughts. This finding of greater mPFC activation in BN has also been supported by task‐related fMRI studies investigating body‐related stimuli processing (Spangler & Allen, 2012) and indicates that this pattern of activation is not specifically related to binge eating per se, therefore offering a possible rationale for differences in the psychopathology in BED and BN.

A seemingly contradictory result is the observation of decreased connectivity in the mPFC in patients with BN compared to normal‐weight controls. However, a closer look reveals that patients show decreased activity in the left lateral portion of the PFC. Although little research has been conducted into the functional lateralization of the medial PFC within the DMN, hemispheric lateralization of the mPFC has been traditionally related to emotional control (Davidson, 1998). Specifically, several studies have shown that chronic stress is related to changes in the structural and functional organization of the mPFC. These changes seem to be lateralized with a hyperactivation in the right mPFC (Cerqueira, Almeida, & Sousa, 2008). According to insights in animal research (Costa, Vicente, Cipriano, Miguel, & Nunes‐de‐Souza, 2016; Johnstone, Reekum, Urry, Kalin, & Davidson, 2007), the left mPFC has an inhibitory effect on the right mPFC, thereby controlling stress‐induced anxiety. Previous studies have also observed a strong connection between lesions in the right prefrontal cortex and the development of eating disorders (Houy, Debono, Dechelotte, & Thibaut, 2007; Uher & Treasure, 2005). Potentially, our observation might indicate a failure of the left mPFC to inhibit the right mPFC in BN, contributing to the often observed increased negative emotional processing and higher stress reaction in BN when compared to obese, normal‐weight, and even BED individuals (Peterson et al., 2010).

With regard to the executive network, individuals with BN showed significantly increased activation in the middle frontal gyrus and angular gyrus when compared to the BED group. Functionally connected brain regions of the EN have shown to be strongly coactivated during cognitively challenging tasks and are essential for the active maintenance and manipulation of information in working memory, as well as for judgment and decision‐making in the context of goal‐directed behavior (Menon & Uddin, 2010). Therefore, an increased activity of the EN may indicate altered attentional and cognitive control resources in BN. Potentially, the increased EN activation is related to higher levels of eating restraint (Roberto, Grilo, Masheb, & White, 2010) and reflects a possible means of modulating food and body image‐related anxieties. Interestingly, the analysis of the motor inhibition task performed by the participants indicated a diminished inhibitory motor control in BN patients with high symptom severity, which was reflected in a reduction in frontostriatal activity (Skunde et al., 2016). This observation was limited to neutral stimuli (and not observed with food‐related stimuli), indicating that a general impairment of behavioral inhibition is related to the severity of binge eating symptoms in BN.

The differences in brain networks in individuals with BN, BED, and obesity contribute to the understanding of functional abnormalities on the neuronal level in different eating disorders. Additionally, aberrant brain network functioning may constitute a biological marker of eating disorders and inform the development of new treatment strategies. As the degree of connectivity between the dACC and retrosplenial cortex is related to the symptomology of BN, these markers (on the neuronal as well as behavioral level) can be used as potential prognostic or diagnostic markers. In fact, applying techniques such as machine vector learning or multivariate pattern analysis may allow for a better characterization of brain network abnormalities as disorder‐specific neural correlates (Haynes & Rees, 2006; Norman, Polyn, Detre, & Haxby, 2006). Additionally, examining brain network connectivity before and after treatment can inform the understanding of the underlying abnormalities contributing to the maintenance of eating disorders. Given that BED and BN have partially overlapping symptoms and the observation that patients may move between disorders, studying symptom‐related neural functioning could elucidate the neurobiological diversity involved in eating disorders and therefore allow for a better distinction and characterization of patients.

Even though different psychiatric disorders are associated with unique neural correlates, structural and functional abnormalities in the salience network have been consistently reported (Downar, Blumberger, & Daskalakis, 2016). Specifically, aberrant functioning of the SN has been observed in major depressive disorder (Manoliu et al., 2013), posttraumatic stress disorder (Lei et al., 2015), obsessive–compulsive disorder (Posner et al., 2017), and schizophrenia (Palaniyappan, White, & Liddle, 2012). Thus, deficiencies in the integration of sensory, emotional, and cognitive cues as well as response selection might be shared neural basis throughout the spectrum of psychiatric illnesses. Resting‐state fMRI can further be used to develop brain‐based treatment frameworks targeting this common neural substrate. Furthermore, previous studies have investigated the feasibility of brain stimulation techniques targeting the SN, observing symptom improvement in different psychiatric disorders following the stimulation of the dACC (Carmi et al., 2018; Hayward et al., 2007; Kreuzer et al., 2015; Vanneste, Ost, Langguth, & Ridder, 2014). A common target site for therapeutic repetitive transcranial magnetic stimulation (rTMS) is the prefrontal cortex (Fitzgerald, Maller, Hoy, Thomson, & Daskalakis, 2009). Interestingly, a study by Dunlop et al (2015) could find a beneficial effect of rTMS on the dmPFC in the frequency of binge and purge behavior in individuals with BN. Our findings further emphasize the importance of focality with regard to the lateralization and precision (i.e., ventral and dorsal) of the mPFC.

There are several limitations to this study. There were four male participants in the BED and their respective overweight control group, which makes comparisons between the BN and BED group difficult. Neither normal‐weight individuals with BED nor overweight individuals with BN were included in our sample. Since binge eating may be a primary symptom leading to overweight and obesity, it is therefore important for future studies to investigate BED in nonobese individuals. In fact, previous research indicates that normal‐weight individuals with BED differ in their weight control behavior compared to participants with obesity and BED (Goldschmidt et al., 2011). This difference raises the issue of classifying study participants by diagnostic groups. More information may be gained from studying symptom‐related functional brain alterations rather than classification by diagnosis. Thus, there is a need for additional research to investigate the overlap and mixture of symptoms between bulimic‐type eating disorders. Furthermore, there were significant differences regarding age, BMI, and amount of binge episodes between patients. These differences may have confounded our results; further studies should investigate matched groups. Moreover, the observed functional connectivity differences might have been influenced by structural abnormalities frequently underlying eating disorders (Raji et al., 2010; Ward, Carlsson, Trivedi, Sager, & Johnson, 2005; Yau, Castro, Tagani, Tsui, & Convit, 2012). Finally, longitudinal studies are needed in order to decipher the causal and temporal role of functional abnormalities present in eating disorders.

In conclusion, this study demonstrates distinct functional connectivity patterns in bulimic‐type eating disorders. We observed aberrant functional connectivity within both the salience‐ and default mode network in our patient groups, indicating the importance of brain structures subserving emotional, arousal, cognitive control, salience attribution and self‐referential processing in bulimic‐type eating disorders. Furthermore, despite a significant overlap of symptomatology, our results hint to neurobiological differences between BN and BED and put emphasis on the importance of defining bulimic‐type eating disorders along a continuum rather than as a set of categories.
