# Supplementary material for: Altered functional connectivity in binge eating disorder and bulimia nervosa: A resting‐state fMRI study
Source: Brain Behav. 2019 Jan 15;9(2):e01207. doi: 10.1002/brb3.1207 (PMC6379643; doi:10.1002/brb3.1207)
Supplement: Supplementary file 1 [file BRB3-9-e01207-s001.docx]

**Supplementary Material**

**Table S1:** Results obtained from the independent component analysis comparing both control and BTE groups pooled together

| **Group/Brain regions** | **Hemisphere** | **BA** | ***x*** | ***y*** | ***z*** | **No. of voxels** | ***p-*value** | ***t-*value** |
| --- | --- | --- | --- | --- | --- | --- | --- | --- |
| ***Salience network*** |  |  |  |  |  |  |  |  |
| ***CON > BTE*** |  |  |  |  |  |  |  |  |
| Medial dorsal CC (anterior) | L | 32 | 0 | 30 | 24 | 43 | .01 | 4.61 |
| ***Executive network*** |  |  |  |  |  |  |  |  |
| ***CON > BTE*** |  |  |  |  |  |  |  |  |
| Inferior Parietal | L | 40 | -39 | -57 | 51 | 58 | .003 | 4.89 |
|  |  |  |  |  |  |  |  |  |
|  |  |  |  |  |  |  |  |  |

Results were FWE cluster-corrected (voxel *p* < .001; cluster > 30 voxels, *p* < .05), *n* = 25. CON contains ConBED and ConBN pooled; BTE contains BED and BN pooled. BA = Brodmann area; L = left; R = right; CC = cingulate cortex.
